# Improvement of the Quality of Wild Rocket (*Diplotaxis tenuifolia*) with Respect to Health-Related Compounds by Enhanced Growth Irradiance

**DOI:** 10.1021/acs.jafc.3c07698

**Published:** 2024-04-22

**Authors:** Fahimeh Khoramizadeh, Adriana Garibay-Hernández, Hans-Peter Mock, Wolfgang Bilger

**Affiliations:** † Botanical Institute, Christian-Albrechts University Kiel, Olshausenstr. 40, Kiel D-24098, Germany; ‡ Molecular Biotechnology and Systems Biology, Rheinland-Pfälzische TU Kaiserslautern, Paul-Ehrlich Straße 23, Kaiserslautern D-67663, Germany; § Leibniz Institute for Plant Genetics and Crop Plant Research (IPK), Corrensstraße 3, Seeland, OT Gatersleben D-06466, Germany

**Keywords:** α-tocopherol, flavonoids, zeaxanthin, epidermal UV transmittance, leafy greens

## Abstract

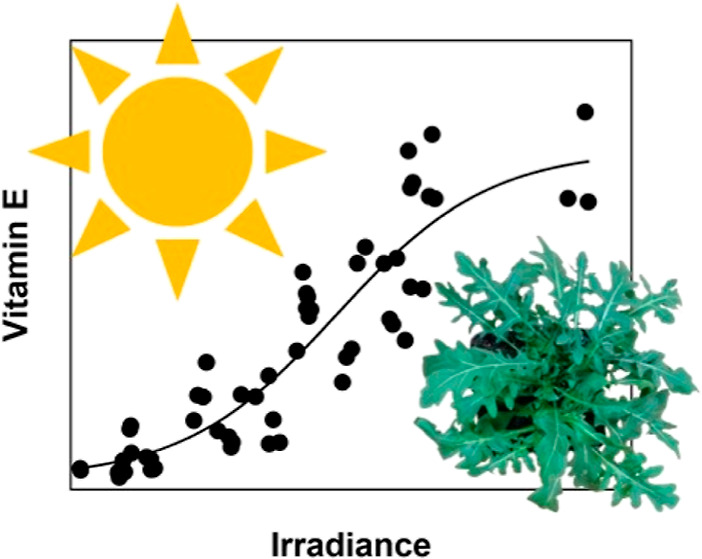

For healthier human nutrition, it is desirable to provide food with a high content of nutraceuticals such as polyphenolics, vitamins, and carotenoids. We investigated to what extent high growth irradiance influences the content of phenolics, α-tocopherol and carotenoids, in wild rocket (*Diplotaxis tenuifolia*), which is increasingly used as a salad green. Potted plants were grown in a climate chamber with a 16 h day length at photosynthetic photon flux densities varying from 20 to 1250 μmol m^–2^ s^–1^. Measurements of the maximal quantum yield of photosystem II, F_V_/F_M_, and of the epoxidation state of the violaxanthin cycle (V-cycle) showed that the plants did not suffer from excessive light for photosynthesis. Contents of carotenoids belonging to the V-cycle, α-tocopherol and several quercetin derivatives, increased nearly linearly with irradiance. Nonintrusive measurements of chlorophyll fluorescence induced by UV-A and blue light relative to that induced by red light, indicating flavonoid and carotenoid content, allowed not only a semiquantitative measurement of both compounds but also allowed to follow their dynamic changes during reciprocal transfers between low and high growth irradiance. The results show that growth irradiance has a strong influence on the content of three different types of compounds with antioxidative properties and that it is possible to determine the contents of flavonoids and specific carotenoids in intact leaves using chlorophyll fluorescence. The results may be used for breeding to enhance healthy compounds in wild rocket leaves and to monitor their content for selection of appropriate genotypes.

## Introduction

Fruits and vegetables are considered especially healthy because they are a rich source of vitamins and special phytochemicals. Accordingly, a high consumption of fruits and vegetables is desirable for a high uptake of phytonutrients. However, attempts to increase general consumption, such as the campaign of “five a day” had little success.^1^ Besides enhancing their consumption, the improved content of desirable compounds in fruits and vegetables may increase the intake of healthy compounds. However, strategies to enhance the consumption of phytonutrients by enrichment of the nutritional value of plants through genetic manipulation have still limited acceptance in Western countries.^1^ Another avenue to reach this goal is via changes in agricultural practices or traditional breeding. Many of the health-related compounds, such as carotenoids or phenolics, also contribute to the stress resistance of plants since they can serve as antioxidants. Hence, some environmental factors favor the production of phytonutrients. Among them, high light has been proven to enhance phenolics and other antioxidants in many plants (for a review, see Poiroux-Gonord et al., 2010).^1^ Breeding for a higher antioxidant content needs to consider this strong influence of light conditions during growth when the plants are screened for desired genotypes.

A rich source of vitamins and specialized natural compounds is green leafy vegetables. Wild rocket *Diplotaxis tenuifolia* (L.) DC. is a green leafy vegetable belonging to the Brassicaceae family, originating from the Mediterranean regions.^2−4^ It is also known as “rucola”, “arugula”, or “*rucola selvatica*” and in recent years has become more popular than salad rocket (*Eruca sativa (Mill.) Thell.*), which is another species from the Brassicaceae family. Wild rocket is well known for its peppery taste caused by glucosinolates, the precursors of isothiocyanates.^3,5,6^ The cultivation of wild rocket in greenhouses as well as in the field has become increasingly popular in European countries in recent years.^7−9^ It is rich in ascorbic acid, flavonoids, carotenoids, and vitamin E, all of which act as antioxidants in the human body, providing protection against a variety of diseases like cancer, cardiovascular disease, altered blood pressure, and vision problems.^2,10−15^ Plants facing environmental stresses, such as excessive light, drought, salinity, or nutrient deficiency, produce more antioxidant compounds in order to diminish stress caused by reactive oxygen species (ROS).^16−18^ In many higher plant species, excessive excitation of the photosynthetic apparatus at high light is known to increase the content of carotenoids, especially the xanthophylls from the violaxanthin cycle (V-cycle),^19−21^ α-tocopherol,^22^ flavonoids,^23,24^ glucosinolates,^25^ and ascorbic acid.^26^

Antioxidants function in plants through diverse mechanisms. Ascorbic acid detoxifies ROS, especially the hydrogen peroxide produced through the Foyer-Halliwell-Asada cycle in the vicinity of photosystem I (PS I).^26,27^ It is also involved in the reduction of α-tocopheroxyl radicals that result from α-tocopherol oxidation^27,28^ resulting from scavenging ROS in the vicinity of photosystem II (PS II) by α-tocopherol.^29,30^ Carotenoids, especially xanthophylls, are part of the light-harvesting complexes in the photosystems. In the V-cycle, zeaxanthin (Z) is formed from violaxanthin (V) through the intermediate antheraxanthin (A), quickly and reversibly, in high light.^19,31,32^ In addition, as an acclimation to extended high-light exposure, the total pool of intermediates (VAZ) of the V-cycle is increased.^20,32,33^ Besides its photoprotective function by mediating nonradiative dissipation of excessive light,^19,34,35^ zeaxanthin is assumed to support the function of α-tocopherol as a lipid-soluble antioxidant^36^ by stabilizing membrane packing and participating in radical scavenging.^37^ The photoperiod, as well as the irradiance and quality of light, have an effect on the production of flavonoids,^38^ which act as screening compounds against damage induced by UV-radiation and high light in plants.^39^ Among flavonoids, anthocyanin accumulation provides protection against high photosynthetically active irradiance, when the light absorption is beyond the capacity of enzymatic carbon fixation in the Calvin–Benson cycle.^40^ Since light has such a strong effect on the content of leaf antioxidants, manipulating light during plant growth would be an excellent means to enhance the nutritional quality of leaves. However, for practical and economic reasons, the artificial irradiation of crop plants may often not be feasible. Nevertheless, in view of the strong influence of light, it is important to get information on the quantitative relationship among the contents of phytochemicals and light conditions. Awareness of this relationship is also essential when evaluating the success of breeding as plants of different genotypes may have developed at varying irradiance.

To improve the quality of leafy vegetables, it is important to monitor the success of the employed measures rapidly and nonintrusively. Also, in the supply chain, quality control is desirable to detect produce deterioration. For assessing polyphenolic compounds and xanthophylls in intact plant leaves, chlorophyll fluorescence (ChlF) has been applied.^41,42^ This method enables rapid detection and quantification of UV-absorbing compounds such as flavonoids, as well as hydroxycinnamic acids by using commercially available instruments.^43−45^ In this study, we investigated the effect of increasing growth irradiance over a wide range, with photon flux densities (PFDs) between 20 and 1250 μmol m^–2^ s^–1^, on the content of health-related compounds in *D. tenuifolia* leaves. We studied the response of carotenoids, vitamin E, and flavonoids to high light, with a special focus on identifying the phenolic compounds underlying the high irradiance response. Since we increased the irradiance in small steps, we detected polyphasic responses to high light. We also assessed up to which irradiance antioxidant biosynthesis can be enhanced without harming the plants by high-light stress. Finally, we evaluated noninvasive methods to monitor the flavonoid and carotenoid content of wild rocket under changing environmental conditions.

## Materials and Methods

### Plant Material

Seeds of wild rocket (*D. tenuifolia* (L.) DC., “SPERLI’s Rucola”) obtained from Sperli GmbH (Everswinkel, Germany) were sown in a soil substrate (TKS 2, Floragard Vertriebs-GmbH, Oldenburg, Germany). Two plants in each pot (14 cm diameter) were grown in a climate chamber (60% humidity and 21 °C, day and night) under a photoperiod of 16 h per day. Irradiation was provided by ceramic metal halide lamps (CMT360LS WBH EYE, Iwasaki Electric Co., Japan) and irradiance from 400 to 700 nm was determined with a Li-Cor quantum meter (model 185B, Li-Cor, Lincoln, NE) at the surface of the sampled leaves. In order to study the light dependence of secondary metabolites, freshly germinated plants were exposed to different PFD levels, ranging from 20 to 1250 μmol m^–2^ s^–1^ by placing the plants on a ladder with 10 steps in the climate chamber and thereby varying the plant’s distance from the lamps. Due to the different exposure to radiation, the leaf temperature varied between 15 and 28 °C (Figure S1), with an average temperature of 22.6 °C. This experiment was repeated 3 times, with two pots per light level, and at least 5 samples per irradiance level were taken in each experiment. Leaves for sampling were chosen according to their exposure to light, i.e., leaves or parts of leaves shaded by other leaves were not selected. 27–33 days after sowing, samples were taken from the fourth and/or fifth mature leaf between 10:00–13:00 h, which was 4 to 7 h after the light was turned on. For each sample, the irradiance was determined separately at the time of sampling.

For some transfer experiments, leaves were also sampled from potted plants grown at the Botanical Garden at Kiel in June and July 2017. Photosynthetic photon flux density was recorded using a weather station,^46^ and daily light integrals were calculated for the 8 days before sampling.

### Leaf Area and Leaf Mass per Area

The fourth and fifth mature leaves with the same shape and size of 27–28 day-old plants were scanned on a flat-bed scanner (WF- 3640, Epson, Meerbusch, Germany), and their area was determined with Sigma Scan pro 5 (Systat Software, Point Richmond, U.S.A.). Leaf mass per area (LMA) (g m^–2^) was measured on leaf discs (0.64 cm^2^) punched out from the surface of the same leaf, avoiding the central vein. For each leaf, 5 to 15 discs were punched. The total fresh weight of the leaf discs was determined before the discs were dried at 60 °C in an oven (Memmert, Schwabach, Germany) for 2 h to reach the dry mass.

### Chlorophyll Fluorescence Measurements

A UVA-PAM fluorometer (Gademann Instruments, Würzburg, Germany) and a Mini-PAM fluorometer (Heinz Walz GmbH, Effeltrich, Germany) were used to determine ChlF. After 30 to 60 min of dark adaptation, leaves of wild rocket were fixed in a leaf clip (Walz) to apply the three different measuring beams in sequence to the same part of the leaf. The intensity of the measuring beams was weak enough to avoid any actinic effect so that the ChIF was at the F_0_ level. Fluorescence signals were normalized based on the fluorescence signals obtained by using blue and green plastic foils (Walz) as the standards. The ratio of fluorescence of UV-A light [375 nm, F(UV-A)] to the fluorescence of red light [660 nm, F(R)], F(UV-A)/F(R), was used as an indicator of epidermal UV-A screening.^45^ A reference value for 100% epidermal transmittance was obtained by measuring fluorescence from the lower side of low-light-grown wild rocket leaves with removed lower epidermis and used to calculate epidermal UV-A transmittance T(UV-A). Epidermal absorbance as an indicator for UV-A screening pigments was calculated as –log T(UV-A).

The ratio of fluorescence excited by blue light [470 nm, F(B)] to the fluorescence excited by red light [F(R)] was used as a parameter for the relative content of violaxanthin cycle (V-cycle) carotenoids.^41^ This method is based on the competition between chlorophyll and carotenoids for the absorption of blue light and on the lack of energy transfer from the carotenoids of the V-cycle to chlorophyll, especially when these carotenoids are not bound to pigment-protein complexes.^47^ The higher the V-cycle carotenoid content is, the lower the ratio of F(B)/F(R).^41^

### Sampling for Pigment Analysis

For comparison of the nondestructive method with the real pigment content, pigments were analyzed by high-performance liquid chromatography (HPLC). The exact part of the leaf, which was fixed in the leaf clip, was punched out by a cork borer (9 mm diameter) from the leaf avoiding the central vein. Leaf discs were placed in 2 mL Eppendorf tubes containing 5 glass beads (two of 2 mm and three of 4 mm size, Roth GmbH & Co. KG, Karlsruhe, Germany), immediately frozen in liquid nitrogen, and stored at −80 °C in a freezer for further analysis.

### Carotenoid Analysis

The frozen leaf discs were homogenized in their reaction tubes in a Geno Grinder (Type 2000; SPEX CertiPrep, Munich, Germany) for 3 min at 1700 strokes min^–1^. The tubes contained 900 μL of ice-cold 80% (v/v) acetone, prepared by mixing acetone (100%) with 30 mM Tris buffer (pH 7.8; Roth). Samples were incubated for 2 min on a thermoshaker (TSC, Biometra GmbH, Jena, Germany) at 1400 rpm and 4 °C. After centrifugation for 5 min at 14,000 *g* and 4 °C (Biofuge Fresco, Heraeus, Hanau, Germany), the supernatant was collected. The extraction procedure was repeated two times by adding 300 μL of 100% acetone. The combined supernatant (1500 μL) was centrifuged at 4 °C for 10 min at 11,500 g. 500 μL of the extract was filtered through a syringe filter (0.45 μm, Thermo Scientific, Rockwood, U.S.A.) in an HPLC vial and immediately placed in the HPLC autosampler. Quantification of the photosynthetic pigments, carotenoids and chlorophyll, was carried out with an HPLC system of the Agilent 1100-series (Agilent Technologies, Waldbronn, Germany) as described elsewhere.^41^ An exemplary chromatogram is shown in Figure S2. The epoxidation state of the violaxanthin cycle pigments (EPS) was calculated using the equation
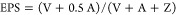


### Tocopherol Analysis

Tocopherol and tocotrienol contents were determined according to the method published in ref (48). 400 μL of *n*-heptane was added to 2 mL Eppendorf tubes containing a frozen leaf disk and glass beads (kept on a precooled aluminum box), before grinding the leaf material in a Geno Grinder (SPEX CertiPrep) for 3 min at 1700 strokes min^–1^. Eppendorf tubes containing the extracts were kept for one night in a −20 °C freezer. The following day, samples were vortexed and centrifuged for 10 min at 16,000 g (Biofuge Fresco), before 100 μL of the extract was added to a microvial for HPLC analysis. Chromatographic analysis of tocopherols was done by injecting 20 μL of extract into a Shimadzu HPLC system equipped with an RF-10A XL fluorescence detector, 10-series (Shimadzu Corporation, Kyoto, Japan). Tocopherol separation was obtained using a LiChrospher Si 60 column (5 μm/250 x 4 mm, Merck, Darmstadt, Germany) and an isocratic system with a flow of 1 mLmin^–1^ of the eluent [*n*-heptane and isopropanol (99/1, v/v)]. Four different kinds of tocopherols and tocotrienols (α-, β-, γ-, and δ-tocopherol, respectively) were detected by their fluorescence at 328 nm after excitation at 290 nm. Peaks were identified based on their retention time in comparison with standards (Merck KGaA, Darmstadt, Germany). Tocopherol standards were measured at the beginning, after every five samples, and at the end of the analysis.

### Flavonoid Analysis

Extraction and HPLC analysis of polyphenolic compounds were done as described before^41^ with the exception that 50% v/v methanol acidified with 1% v/v formic acid (Panreac, AppliChem GmbH, Darmstadt, Germany) was used as the extraction solvent. Flavonoids were detected at 360 nm using a photodiode array detector (PDA) and identified based on their absorption spectra after comparison with HPLC-MS (see below). Anthocyanin detection was performed at 520 nm.

### Identification of Major Soluble Phenolics via Mass Spectrometry

The methanolic extracts for flavonoid analysis (described above) were used to annotate the major soluble phenolics (mostly flavonoids). Vacuum-dried extracts were resuspended in 80% (v/v) methanol using a volume that corresponded to a 5-fold concentration factor of the original volume (e.g., a 500 μL vacuum-dried extract was resuspended in 100 μL). Samples were vortexed (20 s), agitated (4 °C, 20 min, 12,000 rpm), and stored at −20 °C for further analysis. For compound identification, the samples were analyzed via RP-UPLC-PDA-ESI-UHR-QTOF-MS/MS (reversed phase-ultraperformance liquid chromatography-photo diode array-electrospray ionization-ultrahigh-resolution-quadrupole time of flight-tandem mass spectrometry). Before analysis, aliquots from the concentrated extracts were mixed in a ratio of 4:1 (v/v) with 0.5% v/v formic acid, incubated overnight (−20 °C), and centrifuged (22,500 g, 7 °C, 10 min) to remove precipitates. The analysis was carried out using an Acquity UPLC system (Waters, Germany), equipped with an Acquity PDA eλ detector, coupled to a maXis Impact ESI-QTOF MS (Bruker Daltonik GmbH, Germany) with an injection volume of 5 μL. The separation was done in an Acquity UPLC CSH Phenyl-Hexyl (1.7 μm, 2.1 × 100 mm; Waters, Germany) column coupled to an Acquity UPLC CSH Phenyl-Hexyl VanGuard (1.7 μm, 2.1 × 5 mm; Waters, Germany) precolumn, at 40 °C and 0.4 mL min^–1^, using the following gradient (eluent A, 0.1% v/v formic acid in water; B, 0.1% v/v formic acid in acetonitrile): from 5 to 20% B for 4.5 min, from 20 to 45% B from 4.5 to 9 min, from 45 to 100% B from 9 to 11 min, and an isocratic hold from 11 to 12.5 min to clean the column; after each run, the column was equilibrated to the starting conditions (95% A, 5% B). PDA detection was performed in a range between 210 and 800 nm at a resolution of 1.2 nm and a sampling rate of 20 points s^–1^. A fraction (83%) of the eluate outlet from the PDA detector was coupled to the ESI source. MS analyses were performed in positive ionization mode using the following parameters: 50–1500 *m*/*z*; capillary voltage: 4 kV; nebulizer: 3 bar; dry gas: 9.6 L min-1; dry temperature: 220 °C; hexapole radiofrequency (RF) voltage: 100 V peak-to-peak (Vpp); funnel 1 RF: 300 Vpp; funnel 2 RF: 600 Vpp; prepulse storage time: 13 μs; transfer time: 100 μs; low mass: 80 *m*/*z*; collision cell RF: 800 Vpp; collision energy: 8 eV. Tandem MS was done in auto MS/MS mode using collision-induced dissociation with the following settings: absolute area threshold: 5000 counts; exclusion activation: 15 spectra; exclusion release: 30 s; collision energy values (*z* = 1, 2, 3; isolation mass = 500; width = 8): 35, 25, 20 eV; collision energy values (*z* = 1, 2, 3; isolation mass = 1000; width = 10): 50, 40, 35 eV. The system was calibrated before each run with 10 mM sodium formate (water/isopropanol 1:1 v/v) at an infusion flow rate of 0.12 mL h^–1^, using an enhanced quadratic calibration mode. The Compass HyStar 3.2 SR2 software (Bruker Daltonik GmbH) was used to operate and coordinate LC-PDA-MS data acquisition. Data processing, analysis, and compound identification were performed using the software packages Compass Data Analysis V4.4 and MetaboScape 5.0 (Bruker Daltonik GmbH, Germany). Compound identity was confirmed by exact mass (error <1 ppm), isotopic pattern, MS/MS fragmentation, and PDA spectra (Table S1). Commercial standards were employed when they were available. The major phenolics identified via LC–PDA–MS analysis were matched to those detected by HPLC analysis based on their absorption spectra.

### Photosystem II Quantum Efficiency

The maximal quantum efficiency of PSII, determined as F_V_/F_M_, was measured with an Imaging-PAM fluorometer (Walz) on intact plants after a predarkening time of 20 to 30 min.

### Statistical Analysis

Outliers were detected by the Grubbs’ test method, which is also called the ESD method (extreme studentized deviate). All statistical analyses and regressions were calculated using SigmaPlot 13. Regressions were calculated according to the best fit using mostly linear or hyperbolic relationships, which are indicated in the figure captions. However, mathematical models should not be used to deduce functional mechanisms.

## Results

### Growth Irradiance Effects on Leaf Morphology

**Figure 1:**
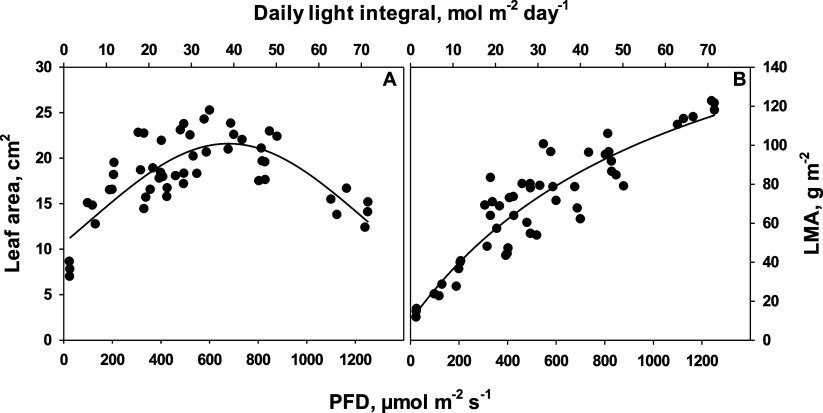
(A) Wild rocket leaf size and (B) LMA as a function of the growth irradiance [determined as (PFD, 400–700 nm, lower *x*-axis) or daily integral of PFD, upper *x*-axis]. All data points represent the area of single fourth or fifth mature rosette leaves. Samples were taken between 4 and 7 h after light was turned on. Lines were drawn by regression, using a Gaussian equation with three parameters for leaf area (*r*^2^ = 0.636) and a rectangular hyperbola with three parameters for LMA (*r*^2^ = 0.846).

The size and mass of the selected wild rocket leaves changed with the different growth irradiances. Leaf area increased with increasing irradiance to a broad maximum at around 600 μmol m^–2^ s^–1^ and declined at higher irradiances (Figure 1A). In contrast, the LMA increased continuously over the whole irradiance range (Figure 1B).

### Light-Dependent Formation of Flavonoid Compounds

The soluble phenolics of the wild rocket leaves were quantified by HPLC equipped with diode array detection (HPLC-PDA). Through further UPLC-PDA-MS analysis of selected samples, we identified phenolic compounds in the wild rocket leaves responsible for more than 90% of the total peak area at 360 nm in the chromatograms. For this purpose, we analyzed the UV-Vis absorbance spectra, retention times, and relative abundance of the major compounds in the HPLC-PDA data and compared them to those detected via UPLC-PDA-MS. A detailed description of the identified compounds is given in Table S1.

**Figure 2:**
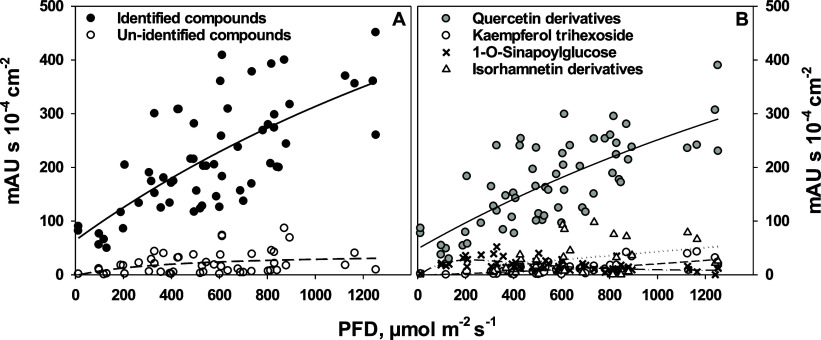
(A) HPLC–PDA peak areas at a detection wavelength of 360 nm of identified (without cyanidin derivatives; closed circles, solid line) and unidentified soluble phenolic compounds (open circles, dashed line) as a function of growth irradiance. (B) HPLC peak areas at 360 nm of the identified phenolics, grouped according to their compound class, as a function of growth irradiance: quercetin derivatives (gray circles, solid line), kaempferol derivatives (open circles, dashed line), 1-*O*-sinapoyl glucose (crosses, dash-dotted line), and isorhamnetin derivatives (open triangles, dotted line). Each point represents the extract of a single leaf. The lines were drawn by regression, using a rectangular hyperbola (identified compounds, *r*^2^ = 0.51, unidentified compounds, *r*^2^ = 0.11, and quercetin derivatives, *r*^2^ = 0.56), exponential growth with three parameters (kaempferol trihexoside, *r*^2^ = 0.48), double exponential decay with five parameters (1-*O*-sinapoyl glucose, *r*^2^ = 0.38), and linear regression (isorhamnetin derivatives, *r*^2^ = 0.11).

The content of soluble phenolics in wild rocket leaves increased in a light-dependent manner (Figure 2A) and was dominated by quercetin derivatives (Figure 2B). Of the five identified quercetin derivatives, three of them, quercetin 3,3′,4′-trihexoside, quercetin 3,4‘-dihexoside-3‘-(6-sinapoyl-hexoside), and quercetin 3-(2-sinapoyl-hexoside)-3‘-(6-sinapoyl-hexoside)-4‘-hexoside, were the major compounds, whereas quercetin 3-glucoside and quercetin 3-(2-feruloyl-hexoside)-3‘-(6-sinapoyl-hexoside)-4‘-hexoside contributed on average to 4% (SD 4.3%) of the phenolics content (Figure S3). The amounts of other observed flavonoids, such as kaempferol-3-dihexoside-7-hexoside, isorhamnetin-3-glucoside, isorhamnetin-3,4′-dihexoside, and isorhamnetin-3,4′-dihexoside hexoside, also increased with growth irradiance but to a smaller extent than the quercetin derivatives. The content of the hydroxycinnamic acid, 1*-O*-sinapoyl glucose, increased to a maximum PFD of 300 μmol m^–2^ s^–1^ and declined again at higher irradiances (Figures 2B and S3B). From PFDs of 400 μmol m^–2^ s^–1^ upward, we detected an anthocyanin identified as a cyanidin-malonyl-hexoside derivative.

### Photosynthesis-Related Parameters and Light-Dependent Response of Lipophilic Antioxidants

**Figure 3:**
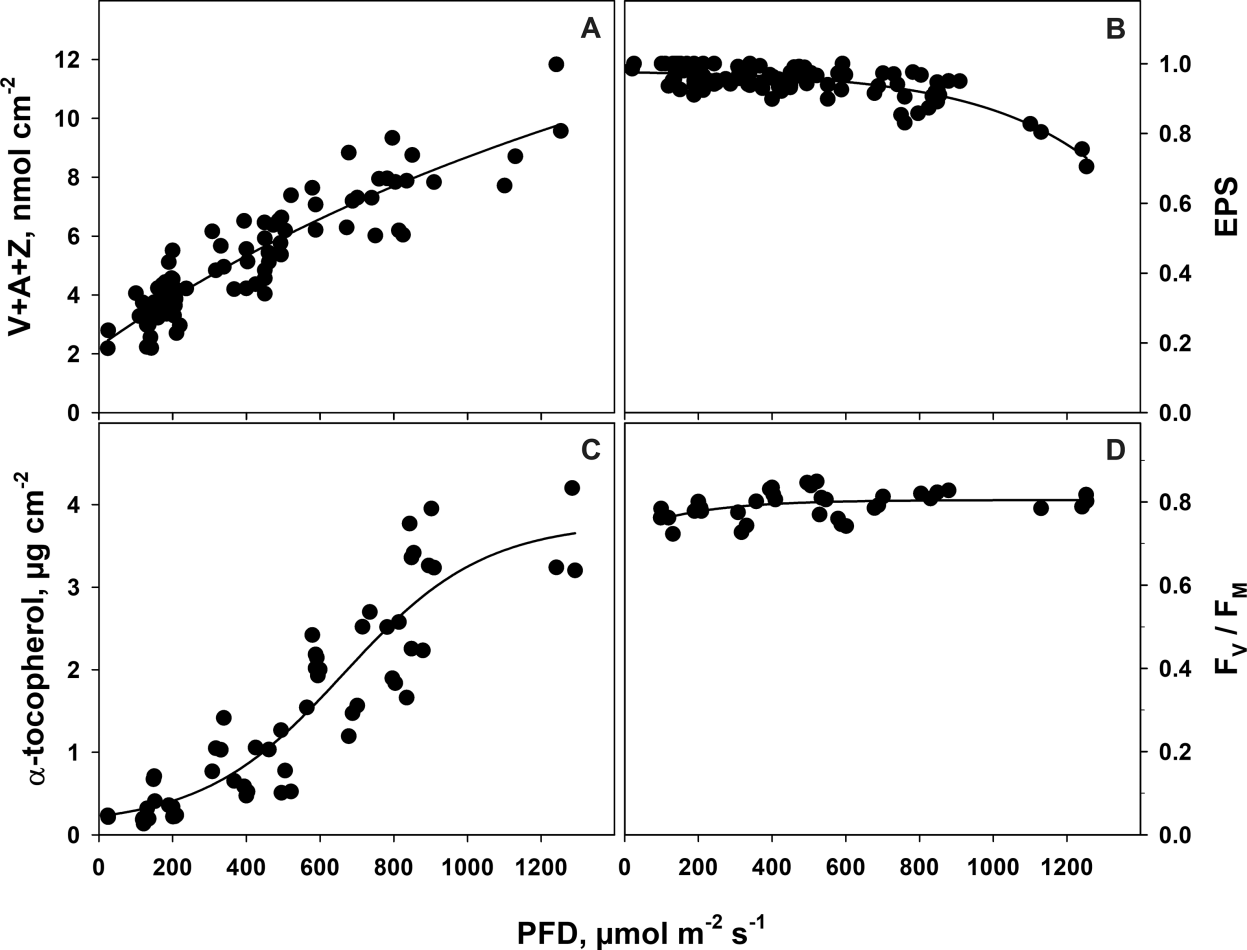
(A) Content of the V-cycle pool size (V + A + Z) per leaf area and (B) its epoxidation state (EPS), (C) content of α-tocopherol, and (D) quantum efficiency of PS II (F_V_/F_M_) as a function of incident PFD. Each data point represents a fourth or fifth mature leaf. F_V_/F_M_ was determined only in one out of the three experiments shown in panels A–C. Lines were drawn by regression using a sigmoidal function with four parameters (A, *r*^2^ = 0.83), exponential rise to maximum with three parameters (after temporary inversion of the *x*-axis) (B, *r*^2^ = 0.65), a rectangular hyperbola with three parameters (C, *r*^2^ = 0.83), and an exponential rise to a maximum (D, *r*^2^ = 0.18).

With increasing irradiance, the pool size of the V-cycle pigments increased linearly, irrespective of relating their content to the leaf area (Figure 3A) or chlorophyll content (Figure S4). The V-cycle pool in wild rocket grown at irradiances around 1200 μmol m^–2^ s^–1^ was four times higher than in leaves grown at the lowest irradiance of 20 μmol m^–2^ s^–1^. The content of the lipophilic antioxidant zeaxanthin, as reflected by the high epoxidation state (EPS) of the V-cycle (Figure 3B), remained low up to a PFD of 900 μmol m^–2^ s^–1^, which indicates that photosynthesis could fully acclimate to the irradiance in this range.

The content of another lipophilic antioxidant, α-tocopherol, showed a strong dependency on irradiance (Figure 3C). Leaves grown at the highest irradiances contained up to 9-fold more α-tocopherol than leaves grown at the lowest irradiances. Although the decline in EPS indicated high-light stress at PFDs above 1000 μmol m^–2^ s^–1^, wild rocket leaves could apparently regulate oxidative stress and remained healthy. This is demonstrated by the quantum efficiencies of PS II (F_V_/F_M_), which did not fall below 0.79 even at a growth irradiance of 1250 μmol m^–2^s^–1^ (Figure 3D).

### Noninvasive Detection of Carotenoid and Flavonoid Compounds

**Figure 4:**
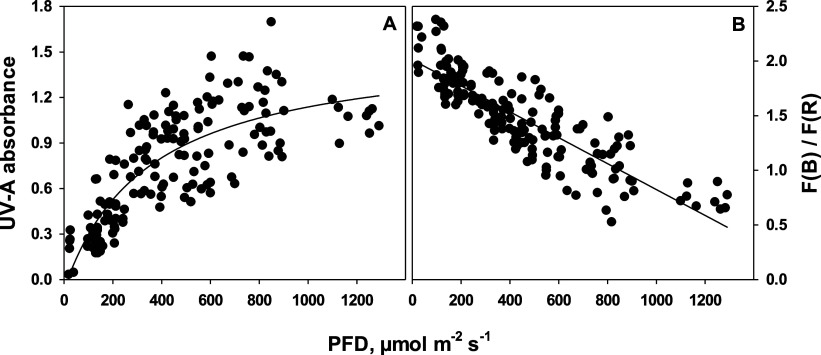
(A) Dependency of epidermal UV-A absorbance as a measure of flavonoid content and (B) dependency of the ratio of the blue-light- to red-light-induced fluorescence, F(B)/F(R), as a measure of V-cycle pigment content on incident PFD. The lines were drawn by regression, using a rectangular hyperbola with three parameters (A, *r*^2^ = 0.65) and linear regression (B, *r*^2^ = 0.74).

Previously, it has been shown that the content of epidermally located UV-absorbing compounds, such as phenolics, can be followed nonintrusively in intact leaves by measuring ChlF.^42,49,50^ Similarly, changes in carotenoid content, predominantly caused by changes in the VAZ pool, have been shown to result in a changed ratio of blue to red excited ChlF, [F(B)/F(R)].^41^ Apparent epidermal UV-A absorbance, calculated from ChlF measurements, increased in wild rocket leaves with irradiance, saturating at high light at absorbance values of around 1.3 (Figure 4A). The F(B)/F(R) ratio decreased linearly with increasing irradiances (Figure 4B).

**Figure 5:**
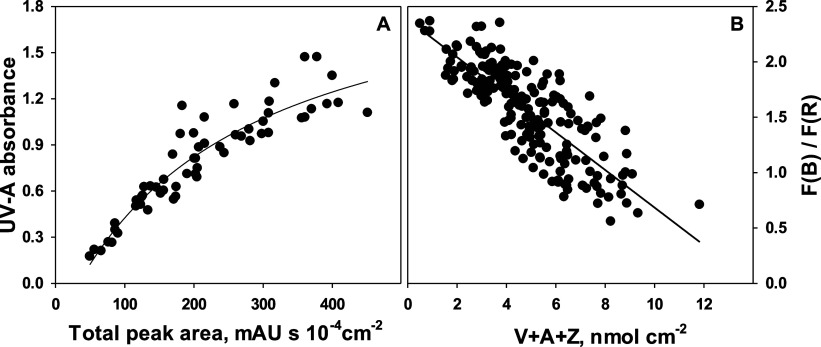
(A) UV-A absorbance as a function of phenolic content expressed as total HPLC–PDA peak area at 360 nm and (B) ratio of F(B)/F(R) as a function of the V-cycle pool per area for wild rocket leaves grown at a wide range of irradiances (20–1250 μmol m^–2^ s^–1^). Each data point represents a single leaf sample from 2 (A) or 16 (B) independent experiments. The lines were drawn by regression, using a rectangular hyperbola with three parameters (A, *r*^2^ = 0.86) and linear regression (B, *r*^2^ = 0.66).

**Figure 6:**
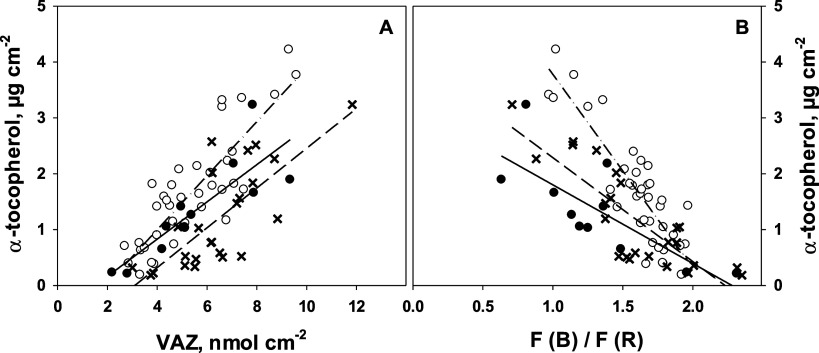
(A) α-tocopherol as a function of the area-related V-cycle pool size and (B) as a function of the fluorescence excitation ratio F(B)/F(R) measured from each single leaf. Results are from three different experiments: experiment 1, black circles, solid lines, *r*^2^ = 0.71 and 0.58 in panels A and B, respectively; experiment 2, open circles, dash-dotted lines, *r*^2^ = 0.72 and 0.76, respectively; experiment three, crosses, dashed lines, *r*^2^ = 0.56 and 0.69, respectively. Each data point represents a single leaf.

As already expected from the irradiance dependency of the optical parameters, both the UV-A absorbance and the F(B)/F(R) ratio had a tight relationship with flavonoid content and V-cycle pool size, respectively (Figure 5). The relationship was linear for the V-cycle pool size (Figure 5B), while UV-A screening saturated at absorbance values above 1 (Figure 5A). When α-tocopherol and VAZ/area were determined in the same experiment, we detected a correlation between them (*r*^2^ = 0.68). Therefore, a linear correlation (*r*^2^ = 0.71) was also found between α-tocopherol and the optical parameter F(B)/F(R) (Figure 6B). Hence, under the given conditions, this parameter could be used as a proxy for α-tocopherol, even though there is no direct functional relationship between both.

**Figure 7:**
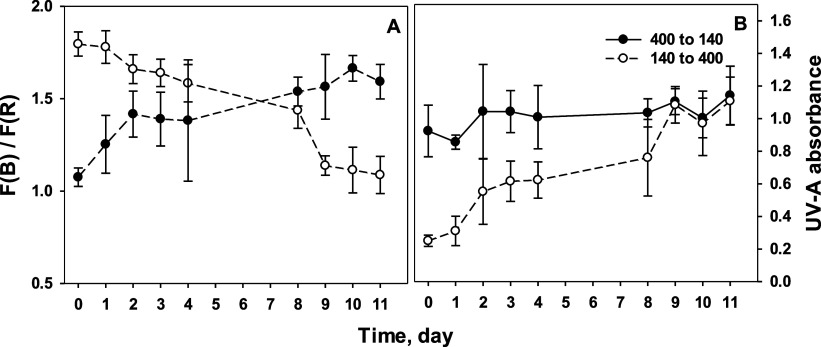
(A) Dynamics of the ratio of F(B)/F(R) as a rapid indicator of V-cycle pool and (B) UV-A absorbance as a rapid indicator of leaf flavonoid content during 11 days after a step change of the irradiance from 400 to 140 μmol m^–2^ s^–1^ (closed circles) and from 140 to 400 μmol m^–2^s^–1^ (open circles). The data show the mean ± SD from measurements on a total of five different leaves of three plants.

Nonintrusive measurements allow us to follow changes in the detected compounds in the same leaf when environmental conditions change. Therefore, we transferred wild rocket plants grown at low-light to high-light conditions and vice versa and followed the kinetics of the ChlF-based signals. ChlF was measured every day from the same spots on the leaf surfaces. F(B)/F(R) declined over 10 days upon transfer of the plants from the low irradiance to the higher one, whereas the reciprocal transfer resulted in an opposite response (Figure 7A). The final levels of F(B)/F(R) values were the same as or very close to those determined for the origin of the opposite treatment group. While the VAZ pool showed a flexible acclimation in both directions, the flavonoid content (measured as the epidermal UV-A-absorbance) only changed when the irradiance was increased but did not decrease when the plants were transferred to a lower irradiance (Figure 7B).

**Figure 8:**
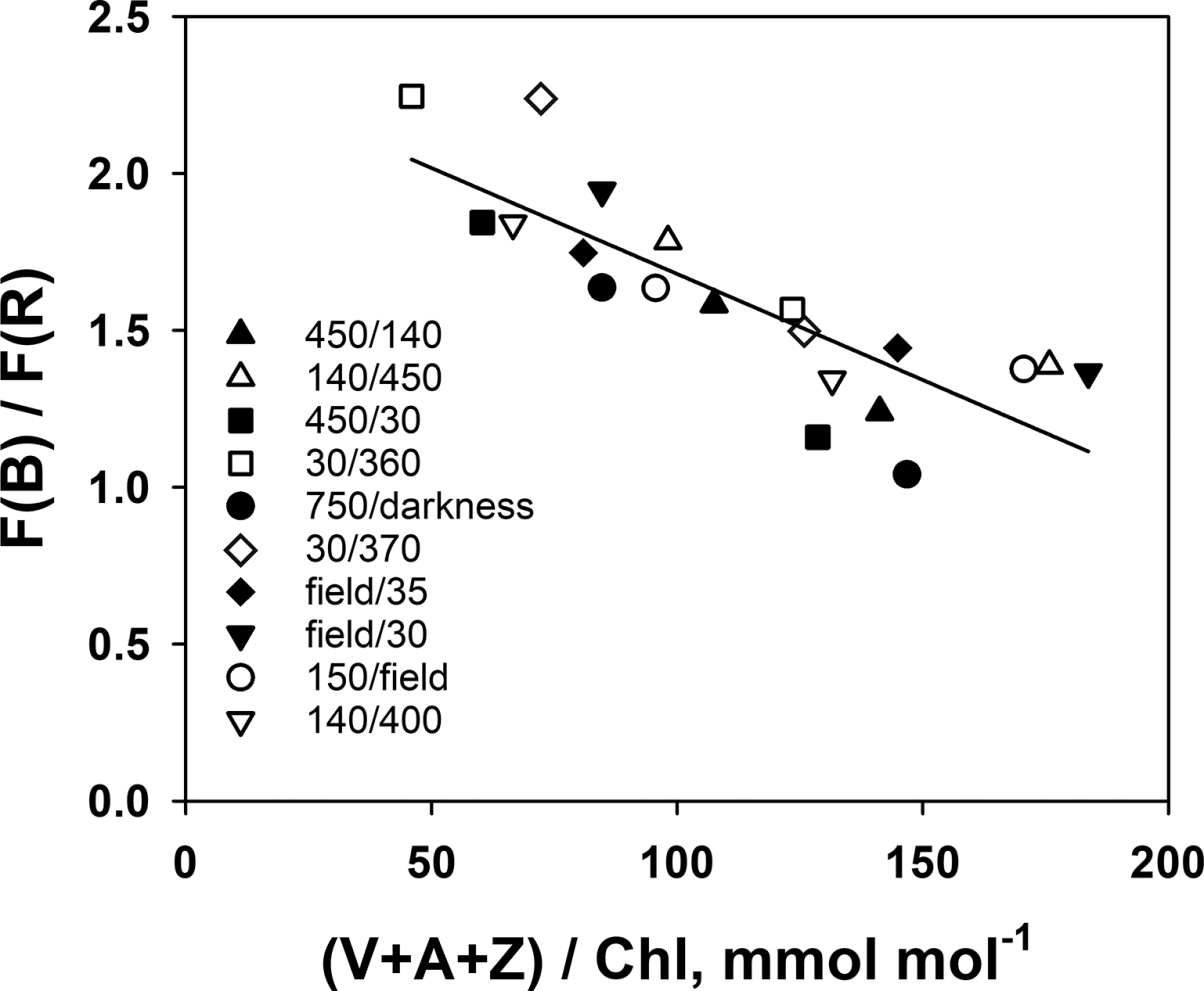
Fluorescence excitation ratio F(B)/F(R) as a function of the V-cycle pool size before and 8 days after the transfer of wild rocket between different irradiances. Data from 10 different irradiance pairs are shown: ▲: 450/140, Δ: 140/450, ■: 450/30, □: 30/360, ●: 750/darkness, ◊: 30/370, ⧫: Field/35, ▼: Field/30, ○:150/Field, open triangle downward: 140/400, where the first number is the original growth irradiance and the second number is the final irradiance. Open symbols denote upshift experiments, and closed symbols denote down-shift experiments. Data points are means of three to five different leaves. Average daily light integral of the preceding 8 days before sampling for the field-grown leaves were 23.0 (⧫), 27.4 (▼), and 27.4 (○) mol m^–2^ day^–1^.

To determine if the F(B)/F(R) ratio did indeed reflect the dynamics of the VAZ pool, samples were taken before the transfer and after 8 days at the new irradiance. Similar transfers as shown in Figure 7 were also made for further irradiance pairs, as indicated in the legend of Figure 8 and always samples taken before transfer and at the end of the observation period. In all experiments, VAZ and F(B)/F(R) followed the same relationship, i.e., when VAZ increased, the fluorescence ratio decreased or vice versa. This led to a negative correlation between both parameters, with an *r*^2^ of 0.64.

## Discussion

In this study, we investigated the possibility of enhancing the content of health-related compounds in wild rocket by growing the plants at a broad range of irradiances. We also evaluated whether it is possible to monitor the content of health-related compounds nonintrusively using ChlF-based measurements. Fully outgrown leaves were sampled at the age of 27–33 d after sowing, with a total average of 30 d, which corresponds to the age used by Bell et al.^25^ The age at which samples are taken varies between 7 and 69 d in the literature, while Bell et al. state that in reality leaves are harvested from plants at an age between 25 and 35 d.^25^

With an increasing growth irradiance, all three investigated compound classes increased strongly. Since leaf temperature also followed the irradiance, higher temperatures might be interpreted as a cause for the increased contents. However, for flavonoids and carotenoids, a temperature dependence opposite to that observed here has been reported. Low temperature enhanced the formation of UV-screening compounds and flavonoids^51,52^ and of V-cycle carotenoids.^53^ On the other hand, high temperature (28 °C) has been reported to repress in *Arabidopsis thaliana* anthocyanin accumulation via the transcription factor HY5.^54^ Hence, the irradiance dependency of the contents of antioxidants could be observed in spite of the mitigating influence of the temperature gradient.

### Carotenoids and α-Tocopherol

In this study, carotenoid content, α-tocopherol, and flavonoids were investigated as major antioxidative phytochemicals in wild rocket. All three groups responded positively in a linear fashion to an increase in growth irradiance. Among the carotenoids, the violaxanthin cycle compounds reacted the most (see also Figure S4). This was to be expected as this reaction has been described many times before for other plant species, though not for wild rocket.^21,55−57^ An increase in the V-cycle pool size has been interpreted as a reaction to irradiance excessive to photosynthesis.^20,58−60^ However, in the conditions applied here, the constantly high EPS up to a PFD of 900 μmol m^–2^ s^–1^ indicates that there was no excessive PFD. Accordingly, the wild rocket was able to adjust the balance between the activities of photosynthetic light and dark reactions successfully. Hence, in this case the regulation of the V-cycle pool size was independent of the existence of excessive irradiance. As a prevention against macular degeneration, a high intake of zeaxanthin and lutein is recommended.^61^ Whereas lutein increased with increasing irradiance (Figure S5), zeaxanthin increased only to a little extent as it is apparent in Figure 3B, which shows that the epoxidation state [equal to (V+0.5A)/(V + A + Z)] remained close to 1 over most PFDs. However, the potential for zeaxanthin formation increased, since the V-cycle pool size increased. It may also occur that less beneficial environmental conditions than those encountered in the growth chamber, as, e.g., nutrient or water deficiency, may cause more zeaxanthin formation. In commercial culture, all handling after harvest happens in low light or darkness in order to minimize water loss via transpiration. Therefore, only by proper pretreatment directly before consumption, zeaxanthin levels could be raised in order to exploit the high potential for zeaxanthin formation in high-light-grown leaves.^21^ Such a pretreatment could be, for example, illumination of leaves with a strong light source giving a PFD of 1000 μmol m^–2^ s^–1^ for 10 min while floating on ice-cold water. The amounts of β-carotene and lutein, the other carotenoid compounds relevant for human nutrition, were around 16 and 20 mg/100 g of fresh weight, respectively, at the highest irradiance (Figure S6). This is more than three times higher than the amount of these compounds reported for *E. sativa*,^62^ a species that is sold as rocket salad.

In contrast to the high number of reports on the response of carotenoids to irradiance, published data specific to α-tocopherol are scarce. A 10-fold higher α-tocopherol content was observed in sun leaves of *Fagus sylvatica* as compared to shade leaves.^63^ Also in *E. sativa*, high irradiance (600 μmol m^–2^s^–1^) treatment caused an increase in the α-tocopherol content,^64^ and in another study, a content of 6.2 mg per 100 g fresh weight has been reported for this species.^62^ When our results were recalculated using fresh weight as a base, an equal amount would be provided by *D. tenuifolia* grown at 600 μmol m^–2^ s^–1^. As the daily intake of α-tocopherol recommended by the World Health Organization (WHO) is 12 mg, consuming around 100 g of the fresh leaves of wild rocket growing at this irradiance would provide almost half of the human diet requirement.^65^

### Soluble Phenolics

Whereas α-tocopherol is a lipophilic antioxidant, flavonoids are mainly glycosylated and, hence, rather hydrophilic. Among all investigated soluble phenolics, the response to increasing irradiances was dominated by quercetin derivatives, which, being *ortho*-dihydroxylated flavonols, are better antioxidants than monohydroxylated flavonols such as kaempferol derivatives.^66,67^ This is in line with other reports^25,68^ where *D. tenuifolia* contained mainly a wide range of quercetin derivatives. However, we and Bell et al. also found kaempferol-3-hexoside,^25^ whereas Taranto et al. observed this compound only in *E. sativa*.^68^ Interestingly, quercetin derivatives responded already at the lowest irradiances, whereas all other flavonoids increased to a much lower extent (Figure 2). Jin et al. reported a 15 times higher content of flavonoids (quercetin, isorhamnetin, and cyanidin) in wild rocket grown at intermediate light (80–120 μmol m^–2^ s^–1^) in comparison to leaves grown at very low light (20–30 μmol m^–2^ s^–1^).^23^ In our work, the content of the only identified hydroxycinnamic acid, sinapoyl glucose, declined at higher irradiances. A decline of HCAs with increasing PFD has been observed before in *Vitis vinifera*.^69^ With the irradiance-dependent increase of α-tocopherol and flavonoids, the content of antioxidant compounds that are healthy for humans could be boosted in wild rocket.

For human consumption, leafy vegetables need to be not only healthy but also tasteful. In wild rocket, the taste is dominated by glucosinolates which were not determined in this study, but it was shown in other studies that their content increases at enhanced irradiance.^25^ Not only taste but also attractiveness and tenderness of the leaves are important criteria for human consumption. With increasing LMA, leaves turn tougher, and above a certain limit, they are no longer desirable for consumption. LMA increased strongly with irradiance (Figure 1). In our lab, we found that leaves sold on the market mostly had an LMA between 20 and 40 g m^–2^, depending on the provider. This would relate to an irradiance of 200 μmol m^–2^ s^–1^ in this work, corresponding to 20% of the maximally possible contents of the V-cycle pool and α-tocopherol. The acceptance by customers of rocket leaves with a higher LMA and presumably a glucosinolate content higher than the one in leaves commonly sold, needs to be tested in the future.

### Noninvasive Monitoring of Phenolics and Carotenoids

In a further approach, we also tested whether rapid nonintrusive methods could assist in the evaluation of the quality of the leaves. Such methods are very helpful for screening in breeding and for quality control in the producer to consumer chain. The need for further breeding of rocket salad has been highlighted by Bell et al.^25^ Screening during breeding can be assisted by optical monitoring. There are several methods based on ChlF, among them the method applied here determining epidermal UV-absorbing compounds by ChlF.^42^ In this work, epidermal UV-A absorbance linearly correlated to total phenolics in leaves grown at irradiances of up to 900 μmol m^–2^ s^–1^. Although this optical method reacts to all compounds absorbing at the wavelength of 375 nm, in the case of wild rocket it would detect primarily quercetin derivatives because they comprised most of the soluble phenolics and their absorption spectra extend the most to long wavelengths of all detected compounds.^70,71^

We observed that UV-A absorbance reacted to a change in irradiance only when it increased but not when it declined. When leaves are harvested for sale, they are kept at low light or in darkness in order to prevent water loss. Since UV-A absorbance will not decrease upon shading and potentially also along the delivery chain, it may be a good indicator of the original growth conditions.

Assessing carotenoid contents in leaves after growth at different irradiances using ChlF has been only recently suggested.^41^ In this former study, only leaves that had already developed at various irradiances were investigated, and hence, the correlation between F(B)/F(R) might have been affected by other irradiance-dependent leaf properties, such as LMA. In our new study, we show for the first time that F(B)/F(R) allows to follow also dynamic changes in the V-cycle pool size in both directions (Figures 7 and 8). Therefore, these data indicate that F(B)/F(R) is not or only negligibly affected by leaf anatomy. This has a bearing on the general usage of this parameter.

Besides monitoring of V-cycle carotenoid content, F(B)/F(R) was correlated to α-tocopherol contents under the given experimental conditions. However, the relationship between both parameters differed between the different experiments. This indicates that additional environmental conditions or specific genotypic properties may affect the relationship between the α-tocopherol and carotenoid contents. This requires further investigation.

The dynamics of the two fluorescence indicators for phenolic compounds and carotenoids were quite different when the irradiance was decreased. While F(UV)/F(R) stayed at a high level, F(B)/F(R) indicated declining carotenoid levels. Hence, the relationship between both indicators may also provide information about the history of light exposure of a leaf in the first days after harvest.

We conclude that the growth irradiance influences the quality of wild rocket leaves, physiologically and phytochemically, by enhancing in an irradiance-dependent manner the content of health-related phytochemicals such as α-tocopherol and flavonoids, which have antioxidative functions. The irradiances used here can probably not be applied cost-efficiently in the commercial cultivation of wild rocket. However, the strong influence of irradiance on contents of antioxidants needs to be considered when different genotypes obtained by breeding are evaluated. Breeding efforts are also supported by phenotyping using the nonintrusive methods applied in this study, which allowed fast detection of the phytochemical quality and to follow the dynamics of health-promoting compounds at pre- and postharvest conditions.

## Supporting Information Available

Data on the temperature conditions during growth, example of an HPLC chromatogram for carotenoid determination, data on light dependencies of single flavonoid compounds and carotenoids, and masses determined for identification of the phenolic compounds (PDF)
