# Supplementary material for: Improvement of the Quality of Wild Rocket (Diplotaxis tenuifolia) with Respect to Health-Related Compounds by Enhanced Growth Irradiance
Source: J Agric Food Chem. 2024 Apr 22;72(17):9735–45. doi: 10.1021/acs.jafc.3c07698 (PMC11066873; doi:10.1021/acs.jafc.3c07698)
Supplement: Supplementary file 1 — jf3c07698_si_001.pdf [file jf3c07698_si_001.pdf]

Supporting information

Improvement of the quality of wild rocket (*Diplotaxis tenuifolia*) with respect to health-related compounds by enhanced growth irradiance

Fahimeh Khoramizadeh [a*], Adriana Garibay-Hernández [b#, c], Hans-Peter Mock [c], Wolfgang Bilger [a]

[a]Botanical Institute, Christian-Albrechts University Kiel, Olshausenstr. 40, D-24098, Kiel, Germany

[b]Molecular Biotechnology and Systems Biology, Rheinland-Pfälzische TU Kaiserslautern, Paul-Ehrlich Straße 23, D-67663, Kaiserslautern, Germany

[c]Leibniz Institute for Plant Genetics and Crop Plant Research (IPK), Corrensstraße 3, D-06466, Seeland, OT Gatersleben, Germany

[*]Email: fkhoramizadeh@bot.uni-kiel.de

Present Address:

[#]A.G.H.: Molecular Biotechnology and Systems Biology, Rheinland-Pfälzische TU, Kaiserslautern, Paul-Ehrlich Straße 23, D-67663, Kaiserslautern, Germany

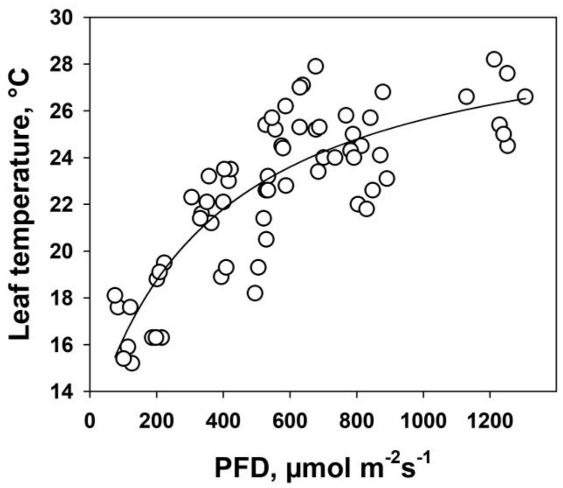

Figure S1. Leaf temperature as determined with an infra-red thermometer as a function of PFD incident on the leaves.

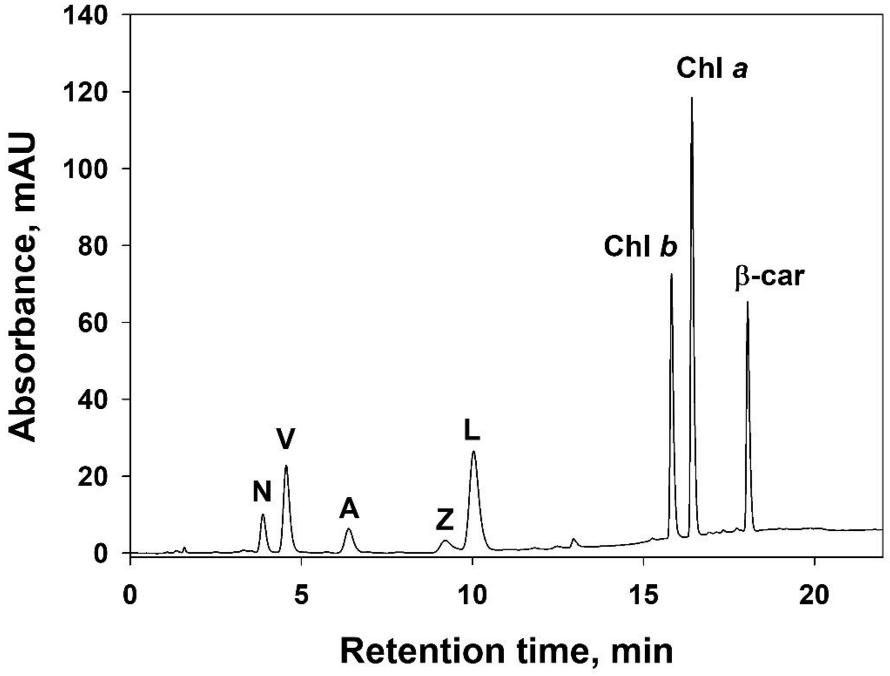

Figure S2. Representative chromatogram of an HPLC analysis for carotenoids and chlorophyll. The analysed sample was from a leaf grown at 1,250 µmol m^{-2} s^{-1}. Detected peaks are labelled, N, neoxanthin, V, violaxanthin, A, antheraxanthin, Z, zeaxanthin, L, lutein, Chl *b*, chlorophyll *b*, Chl *a*, chlorophyll *a*, β-car, β-carotene.

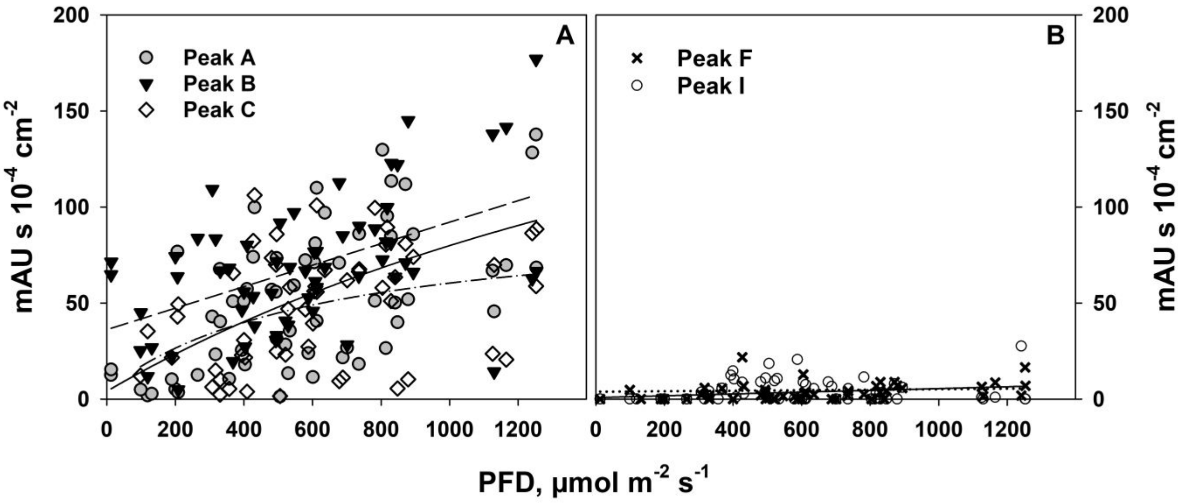

Figure S3. HPLC-PDA peak areas at 360 nm of the quercetin derivatives. (A) peak A, quercetin 3,3',4'-trihexoside (grey circles, solid line), peak B, quercetin 3,4'-dihexoside -3'-(6-sinapoyl- hexoside) (black triangles, dashed line) and peak C, quercetin 3-(2-sinapoyl- hexoside)-3'-(6-sinapoyl- hexoside)-4'-hexoside (open diamonds, dash-dotted line). (B) peak F, quercetin 3-glucoside (crosses, solid line) and peak I, quercetin 3-(2-feruloyl- hexoside)-3'-(6-sinapoyl- hexoside)-4'-hexoside (open circles, dotted line). The lines were drawn by regression, using a rectangularly hyperbolic (peaks A (r^2=0.38) and C (r^2=0.14)) or a linear relationship (peaks B (r^2=0.257), F (r^2=0.10) and I (r^2=0.04)).

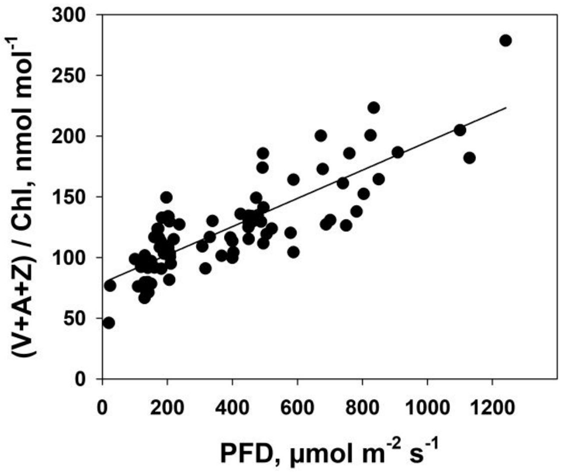

Figure S4. The sum of violaxanthin cycle pigments violaxanthin (V), antheraxanthin (A) and zeaxanthin (Z) expressed in relation to the sum of chlorophyll *a* and *b* as a function of growth irradiance. Each data point represents a single leaf sample. The line was drawn by linear regression (r^2=0.68)

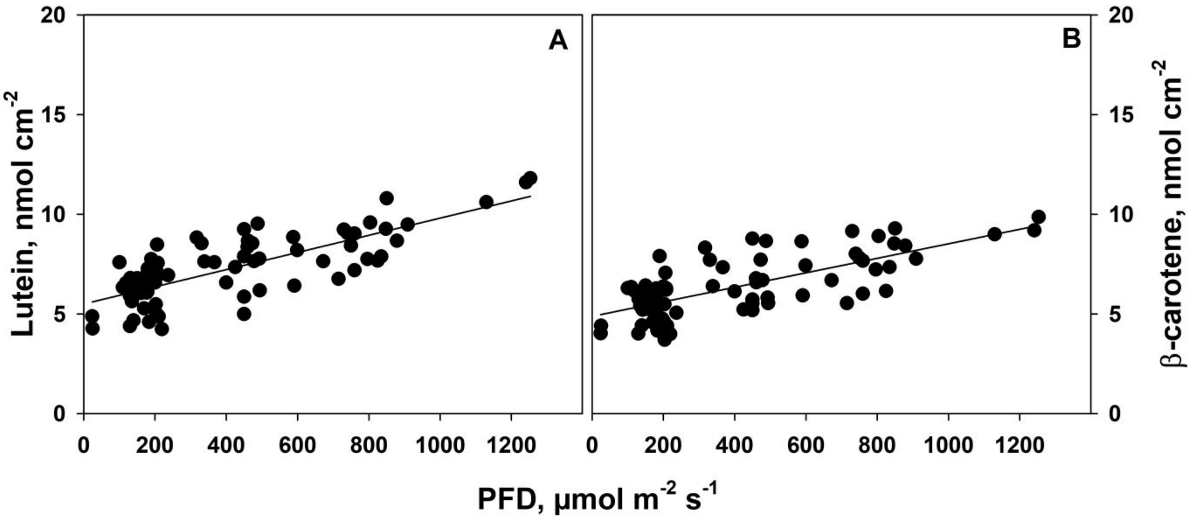

Figure S5. Dependency of the contents per leaf area of the carotenoids lutein (A) and β-carotene (B) on incident photosynthetic photon flux density (PFD). Each data point represents a single leaf sample. The lines were drawn by linear regression (A, r^2=0.59, B, r^2=0.52).

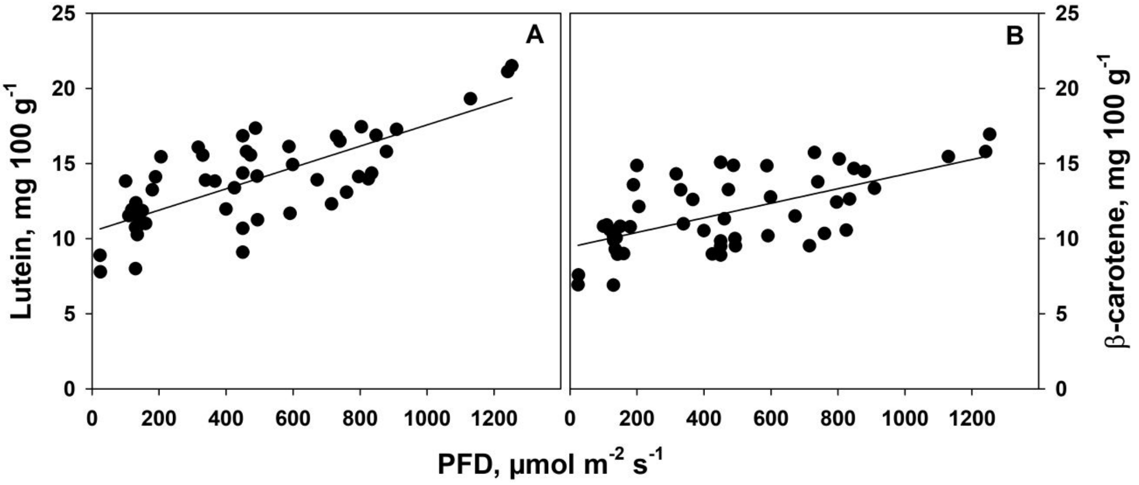

Figure S6. Dependency of the contents per fresh mass of the carotenoids lutein (A) and β-carotene (B) on incident photosynthetic photon flux density (PFD). Each data point represents a single leaf sample. The lines were drawn by linear regression (A, r^2=0.56, B, r^2=0.39).

Table S1. Annotation of major soluble semi-polar phenolic compounds in rucola. Major phenolics were identified by RP-UPLC-PDA-ESI-HR-QTOF-MS/MS. Compound annotations were based on exact mass, isotopic pattern, MS/MS fragmentation, and PDA spectra. The percentage of relative intensities of the fragment ions are indicated in parenthesis. The MS-spectra were compared to the available literature. According to the Metabolomics Standards Initiative recommendations (Ref. 3), most annotations were at the metabolite identification level 2. This means that annotations were based on the physicochemical properties and the spectral similarity with compounds reported in the literature; the specific predicted compounds are indicated in italics. Three compounds were identified at level 1: Rt, MS and PDA spectra confirmed with standards.

Standards: Isorhamnetin 3-glucoside (1228, Extrasynthèse, France); Quercetin 3-glucoside (1119S, Extrasynthèse, France); 1-*O*-sinapoylglucose (isolated by preparative liquid chromatography from cotyledons of *Raphanus sativus*; Ref. 4)

Ant: Anthocyanin; Cya: Cyanidin; Fer: Feruloyl moiety (NL 176.04); Flv: Flavonoid; HCA: Hydroxycinammic acid; Hex: Hexosyl moiety (NL 162.05); Irh: Isorhamnetin; K: Kaempferol; *m/z*: mass to charge ratio; Mal-Hex: Malonyl-hexosyl moiety (NL 248.05); NA: Not Available; NL: Neutral Loss; Que: Quercetin; Rt: retention time; Sin: Sinapoyl moiety (NL 206.05); Sinap: Sinapic acid (NL 224.07 = Sin+H_2O); Std: annotation confirmed with standard.

References:

1. Taranto F, Francese G, Di Dato F, D'Alessandro A, Greco B, Onofaro Sanajà V, Pentagelo A, Mennella G, Tripodi P. 2016. Leaf metabolic, genetic, and morphophysiological profiles of cultivated and wild rocket salad (*Eruca* and *Diplotaxis* Spp.). Journal of Agricultural and Food Chemistry 64: 5824-36.

2. Tohge T, Nishiyama Y, Hirai MY, Yano M, Nakajima JI, Awazuhara M, Inoue E, Takahasi H, Goodnowe D, Kitayama M. 2005. Functional genomics by integrated analysis of metabolome and transcriptome of Arabidopsis plants over-expressing an MYB transcription factor. Plant Journal 42: 218–235.

3. Sumner LW, Amberg A, Barrett D, Beale MH, Beger R, Daykin CA, Fan TWM, Fiehn O, Goodacre R, Griffin JL, Hankemeier T, Hardy N, Harnly J, Higashi R, Kopka J, Lane AN, Lindon JC, Marriott P, Nicholls AW, Reily MD, Thaden JJ, Viant MR (2007) Proposed minimum reporting standards for chemical analysis Chemical Analysis Working Group (CAWG) Metabolomics Standards Initiative (MSI). Metabolomics 3: 211-221.

4. Linscheid M, Wendisch D, Strack D. 1980. The structures of sinapic acid esters and their metabolism in cotyledons of *Raphanus sativus*. Z. Naturforsch. 35c: 907-914.

Peak	Rt *(min)*	Annotation	Agly-cone	λmax *(nm)*	Molecular formula	Monoisotopic mass	Precursor ion	Calculated *(m/z)*	Measured *(m/z)*	Δ ppm	Fragments ions	Measured *(m/z)*	Ref.
A	3.71	Quercetin Hex-Hex-Hex<br>*Quercetin 3,3',4'-triglucoside*	Que *(Flv)*	(s) 266, 336	$C_{33}H_{40}O_{22}$	788.2011	[M+H]$^+$	789.2084	789.2087	0.36	[Que+H+Hex+Hex]$^+$<br>[Que+H+Hex]$^+$<br>[Que+H]$^+$	627.1553 (0.1)<br>465.1032 (73.1)<br>303.0502 (100)	1
B	5.05	Quercetin Hex-Sin-Hex-Hex<br>*Quercetin 3,4'-diglucoside-3'-(6-sinapoyl-glucoside)*	Que *(Flv)*	271, 328	$C_{44}H_{50}O_{26}$	994.2590	[M+H]$^+$	995.2663	995.2668	0.49	[Que+H+Hex+Sin+Hex]$^+$<br>[Que+H+Hex+Sin]$^+$<br>[Que+H+Hex]$^+$<br>[Que+H]$^+$<br>[Sin+H+Hex]$^+$<br>[Sin+H]+	833.1829 (0.1)<br>671.1610 (2.4)<br>465.1029 (37.3)<br>303.0498 (52.2)<br>369.1182 (89.7)<br>207.0705 (100)	1
C	6.17	Quercetin Hex-Sin-Hex-Sin-Hex<br>*Quercetin 3-(2-sinapoyl-glucoside)-3'-(6-sinapoyl-glucoside)-4'-glucoside*	Que *(Flv)*	274, 330	$C_{55}H_{60}O_{30}$	1200.3169	[M+H]$^+$	1201.3242	1201.3243	0.03	[Que+H+Hex+Sin+Hex+Sin]$^+$<br>[Que+H+Hex+Sin+Hex]$^+$<br>[Que+H+Hex+Sin]$^+$<br>[Que+H+Hex]$^+$<br>[Que+H]$^+$<br>[Sin+H+Hex]$^+$<br>[Sin+H]+	1039.2288 (<0.1)<br>833.2097 (<0.1)<br>671.1609 (1.7)<br>465.1033 (5.2)<br>303.0499 (5.3)<br>369.1183 (100)<br>207.0706 (36.3)	1
D	4.54	Kaempferol Hex-Hex-Hex<br>*Kaempferol 3-diglucoside, 7-glucoside*	Kae *(Flv)*	265, 320	$C_{33}H_{40}O_{21}$	772.2062	[M+H]$^+$	773.2135	773.2131	0.50	[Kae+H+Hex]$^+$<br>[Kae+H]$^+$	449.1093 (92.5)<br>287.0546 (100)	1
E	4.65	Isorhamnetin Hex-Hex-Hex<br>*Isorhamnetin 3,4'-diglucoside, -hexoside*	Irh *(Flv*	255, 266, 345	$C_{34}H_{42}O_{22}$	802.2168	[M+H]$^+$	803.2241	803.2241	0.00	[Irh+H+Hex+Hex]$^+$<br>[Irh+H+Hex]$^+$<br>[Irh+H]$^+$	641.1895 (0.3)<br>479.1180 (6.7)<br>317.0658 (100)	1
F	5.48	Quercetin 3-glucoside	Que *(Flv)*	256, (s) 354	$C_{21}H_{20}O_{12}$	464.0955	[M+H]$^+$	465.1027	465.1030	0.63	[Que+H]$^+$	303.0503 (100)	Std; 1
G	5.97	Isorhamnetin Hex-Hex<br>*Isorhamnetin 3,4'-diglucoside*	Irh *(Flv)*	255 (s), (s) 355	$C_{28}H_{32}O_{17}$	640.1639	[M+H]$^+$	641.1712	641.1715	0.49	[Irh+H+Hex]$^+$<br>[Irh+H]$^+$	479.11900 (0.1)<br>317.0662 (100)	1
H	6.1	Isorhamnetin 3-glucoside	Irh *(Flv)*	255 (s), (s) 354	$C_{22}H_{22}O_{12}$	478.1111	[M+H]$^+$	479.1184	479.1186	0.42	[Irh+H]$^+$	317.0666 (100)	Std; 1
I	6.28	Quercetin Hex-Sin-Hex-Fer-Hex<br>*Quercetin 3-(2-feruloyl-glucoside)-3'-(6-sinapoyl-glucoside)-4'-glucoside*	Que *(Flv)*	274, 326	$C_{54}H_{58}O_{29}$	1170.3064	[M+H]$^+$	1171.3136	1171.3131	0.43	[Que+H+Hex+Sin+Hex+Fer]$^+$<br>[Que+H+Hex+Sin]$^+$<br>[Que+H+Hex]$^+$<br>[Que+H]$^+$<br>[Sin+H+Hex]$^+$<br>[Fer+H+Hex]$^+$<br>[Sin+H]$^+$<br>[Fer+H]$^+$	1009.3165 (0.1)<br>671.1598 (2.6)<br>465.1035 (10.0)<br>303.0500 (11.0)<br>369.1180 (27.5)<br>339.1077 (100)<br>207.0700 (26.4)<br>177.0610 (1.1)	1
J	6.01	Cyanidin malonyl-hexoside-derivative	Cya *(Ant)*	328, 537	NA	NA	NA	NA	1241.3181	NA	[M-Mal-Hex]$^+$<br>[Cya+Mal+Hex]$^+$<br>[Cya+Mal+Hex-CO_2]+<br>[Cya]+	993.2681 (12.1)<br>535.1082 (100)<br>491.1186 (5.5)<br>287.0549 (36.2)	2
K	3.36	1-*O*-sinapoylglucose	Sin *(HCA)*	330	$C_{17}H_{22}O_{10}$	386.1213	[2M-CH_2O+Na]$^+$	765.2212	765.2212	0.06	[Sinap+Hex+Na]$^+$<br>[Sinap+Hex-CH_2O+Na]$^+$<br>[Sinap+Na]$^+$<br>[Sinap+H]$^+$<br>[Sin+H]$^+$	409.1101 (100)<br>379.0998 (80.7)<br>247.0548 (7.1)<br>225.0724 (1.9)<br>207.0649 (4.2)	Std

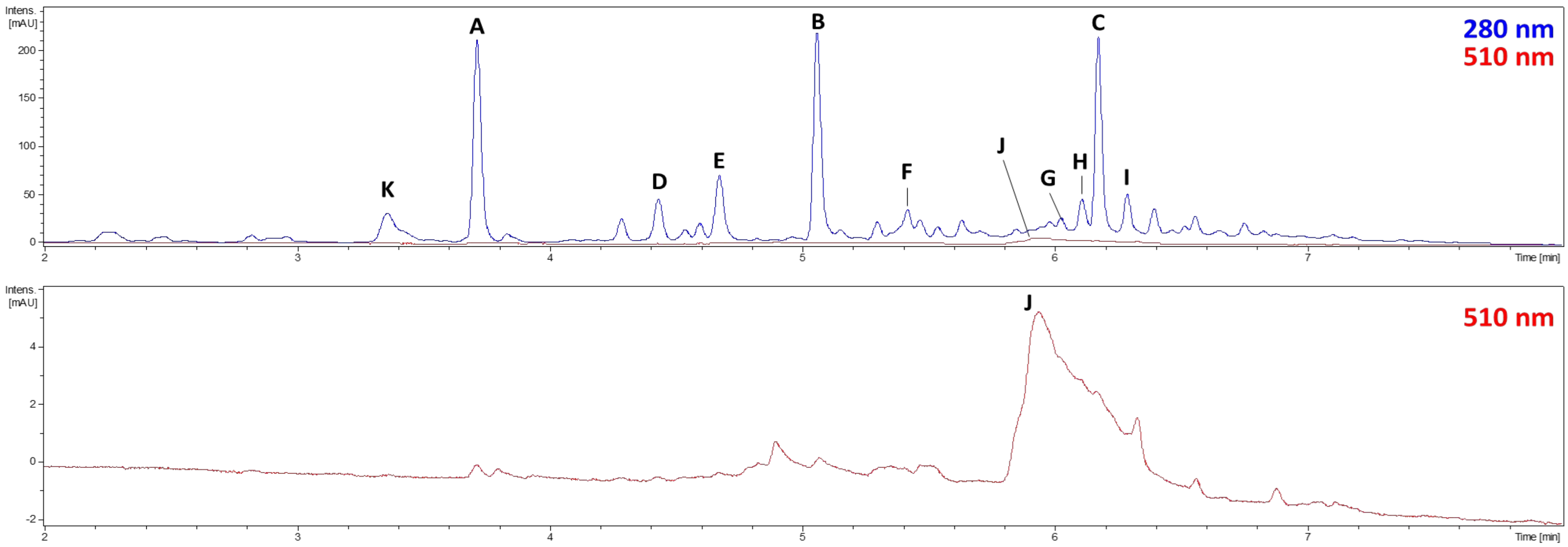

UPLC-PDA-MS chromatogram of UV-absorbing compounds at 280 and 510 nm for a mixed sample of wild rocket leaves from plants grown in a wide range of irradiances (20-1300 μmol m^{-2} s^{-1}).
